# Simulation of between Repeat Variability in Real Time PCR Reactions

**DOI:** 10.1371/journal.pone.0047112

**Published:** 2012-11-26

**Authors:** Antoon Lievens, Stefan Van Aelst, Marc Van den Bulcke, Els Goetghebeur

**Affiliations:** 1 Platform for Molecular Biology and Biotechnology, Scientific Institute of Public Health, Brussels, Belgium; 2 Department of Applied Mathematics and Computer Science, Ghent University, Gent, Belgium; 3 Molecular Biology and Genomics Unit, European Commission - Joint Research Centre, Institute for Health and Consumer Protection, Ispra, Italy; Queen's University Belfast, United Kingdom

## Abstract

While many decisions rely on real time quantitative PCR (qPCR) analysis few attempts have hitherto been made to quantify bounds of precision accounting for the various sources of variation involved in the measurement process. Besides influences of more obvious factors such as camera noise and pipetting variation, changing efficiencies within and between reactions affect PCR results to a degree which is not fully recognized. Here, we develop a statistical framework that models measurement error and other sources of variation as they contribute to fluorescence observations during the amplification process and to derived parameter estimates. Evaluation of reproducibility is then based on simulations capable of generating realistic variation patterns. To this end, we start from a relatively simple statistical model for the evolution of efficiency in a single PCR reaction and introduce additional error components, one at a time, to arrive at stochastic data generation capable of simulating the variation patterns witnessed in repeated reactions (technical repeats). Most of the variation in 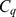 values was adequately captured by the statistical model in terms of foreseen components. To recreate the dispersion of the repeats' plateau levels while keeping the other aspects of the PCR curves within realistic bounds, additional sources of reagent consumption (side reactions) enter into the model. Once an adequate data generating model is available, simulations can serve to evaluate various aspects of PCR under the assumptions of the model and beyond.

## Introduction

Since its inception in the mid 1980s, the polymerase chain reaction (PCR) has revolutionized biomedical research. As little as a single DNA molecule can be specifically amplified to detectable levels. Fluorescent dyes make it possible to monitor this amplification process in real time, allowing relative quantification of the initial amount of template DNA. Due to its unprecedented accuracy and sensitivity, real time quantitative PCR (qPCR) has found widespread application in a wide array of research fields. For a review see [1], [2].

With growing experience, one has recognized that an appreciable degree of uncertainty could accompany stated PCR results. Analysis results are therefore best complemented with an appropriate estimate of precision: an indication of the range within which the true value may be found, given the observations. However, many publications pertaining to real time PCR results forgo uncertainty measures. Although in theory every reaction's outcome should be an exact representation of its initial number of target copies, in practice, several mechanisms introduce variation between repeated reactions (*i.e.* technical repeats: each reaction's volume is pipetted from a single aliquot of reagent mix. Henceforth referred to as ‘repeats’). This variance is not readily explained by measurement error and copy number variation. Even though the use of exponential models is fairly well characterized as a valid approximation to the initial PCR stages of constant and maximal amplification (the so-called ‘exponential phase’), much less is known about the kinetic differences between such repeats as they approach their plateau. Here, we aim to recreate between repeat fluorescence variability by adding probable sources of variation to a statistical model of the PCR process.

The more straightforward models of PCR assume that efficiency (*i.e.* the fold change in target copies after each cycle) is constant during all cycles of the process, or at least up until the quantification cycle (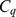, the fractional cycle in which the reaction fluorescence reaches a set threshold). The 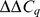 method [3] assumes theoretically maximal efficiency (*i.e.* 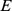 = 2) while others allow for reaction specific efficiencies [4], [5]. Such models seek validity only for a specific region of the reaction (*i.e.* the exponential phase) and have limited use in explaining the underlying processes that drive a PCR reaction towards its plateau.

More detailed models and simulations are available that take the different sub-processes of each cycle of amplification into account (denaturing, annealing, elongation, etc.), either stochastically or deterministically. And although there is a consensus among the majority of these models about the overall inverse-S shaped profile of the efficiency decline [6]–[13], they may differ in the identification of the dominant processes behind the attenuation of efficiency. Some models focus on the thermal inactivation of the polymerase enzyme [14] whereas others argue that this doesn't contribute significantly to the efficiency decline [9], [15]. Others center around saturation of the enzyme activity [7], reagent depletion [6], [10] or primer extension [15]–[17] to model the probability of replication. A number of recent studies point to competition between template-template reannealing and primer-template annealing as the driving force behind efficiency attenuation [9], [11], [13].

Under such a scenario template-template reannealing is initially minimal due to the very high concentration of primers in the mixture. Yet, as primers are consumed and template copies are produced the thermodynamically more favorable reannaeling process starts to dominate over the primer-template hybrid formation. This increasing presence of double stranded DNA (dsDNA) during each successive cycle may cause additional inhibition of the polymerase [18]. Furthermore, as the reaction progresses, the changes in concentration of both primer and template may increase the difference in melting temperature between them [19] which may in turn further promote template-template reannealing [11]. In addition, other processes may contribute to the decrease in reaction efficiency: primer and template damage due to denaturing [14], [20], pyrophosphate poisoning of the polymerase [21], [22], polymerase errors (mutations) [23], [24] and the formation of non-target PCR products [25].

Due to the large number of possible reactions involved and the complexity of the overall process, a bottom-up approach to investigate the leading causes of between-repeat variation was not attempted. While deterministic models are valuable in capturing various detailed specifics of the underlying mechanisms of the PCR process, they lead to approximations of actually observed fluorescence and do not formally account for residual variation. As an alternative, when targeting specific features of the process, we model the fluorescence evolution from a macroscopic perspective, involving global kinetic properties and structured variance components. Formalization of the relationship between the observable variables then allows for inference about the variation of reaction kinetics between repeats. This is accomplished by statistically modeling the efficiency in function of the (baseline subtracted) fluorescence. Initially we will assume that (**A**) the single amplicon fluorescence is constant and that (**B**) reagent consumption due to non-amplification events (so-called side reactions) is negligible, so that the fluorescence is a direct function of the concentrations of both reagents and reaction products. Additional sources of reagent consumption are subsequently brought into the model in order to evaluate their impact on the fluorescence accumulation.

Empirical observations will guide the development of a data generating setup. We start from a large dataset which contains high numbers of repeats of several combinations of reaction conditions (*e.g.* template copies and inhibitor levels). To these data we fit a bilinear model that allows for variable efficiency [26] and then use the observed parameter distributions as the starting point of a simulation approach, allowing to explore the differences between repeats. By adding known and probable sources of variation to the simulation backbone and by exploring their impact on the generated fluorescence curves, an evaluation of the plausible contribution of each source to the total variation is made.

Once such a data generation model is reached, the simulation model will be used to evaluate two aspects of the polymerase chain reactions under the assumptions of the model: **(I)** the number of cycles during which the efficiency is approximately constant, since it is key to 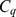-based PCR analysis and **(II)** the position of the second derivative maximum (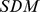) which is often quoted as the end of the exponential phase [27], [28]. Furthermore, the model will be used as a means of inspecting the accuracy and precision of the Full Process Kinetics-PCR (FPK-PCR) parameter estimates through comparison with the simulation input.

## Materials and Methods

The goal of the data generating model is to simulate reactions and their observed variation in fluorescence output by adapting parameter values based on empirical observations. To obtain a realistic set of joint parameter values, a real time PCR dataset was produced from which the model's parameter distributions and responses to changes in initial target copies (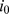) and initial reaction efficiency (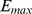) could be estimated. Changes in 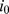 were introduced by varying the input amount of target DNA, changes in 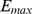 stemmed from adding an inhibitor to the reaction mix.

Practically, a two dimensional array of soybean (*Glycine max*) DNA with initial target concentrations and maximal efficiencies was created: a fourfold dilution series (ranging from approximately 96000 copies to about 375) was run at various inhibitor levels. Inhibitor free reactions were repeated 96 times each, inhibited reactions were repeated 48 times each.

### DNA Samples and PCR reactions

Genetically modified *Glycine max* event GTS-40-3-2 (Roundup Ready Soybean) was grown in house using a growth chamber and standard conditions (25

C, 16 h/8 h day/night regime, 80% humidity, 20,000 lux). Genomic DNA was isolated from leaf tissue using a CTAB based method [29] (all chemicals were obtained from Merck or Acros organics). All DNA extracts were quantified spectrophotometrically (Biorad Smartspec plus). The amount of template copies was calculated from the DNA quantities using haploid genome weights [30].

Inhibited reactions were created by adding isopropanol (Merck), which is a known PCR inhibitor [31], to the reaction mix in various concentrations. A total of 6 different isopropanol conditions were used: 0% (inhibition free), 1%, 1.5%, 2%, 2.5% and 3% (v/v, final concentration). See table 1 for an overview of the resulting 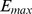 estimates.

**Table 1:** mean 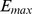 estimate 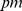 standard deviation as obtained using FPK-PCR for every level of inhibitor and initial template concentration.

	S1	S2	S3	S4	S5
0%	1,89 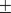 0,02	1,89 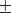 0,01	1,91 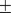 0,01	1,91 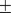 0,02	1,91 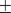 0,02
1%	1,84 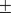 0,01	1,86 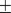 0,02	1,88 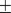 0,01	1,85 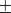 0,03	1,84 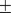 0,02
1,5%	1,85 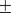 0,01	1,86 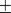 0,02	1,88 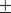 0,01	1,85 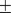 0,03	1,84 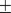 0,02
2%	1,70 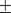 0,04	1,72 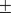 0,03	1,71 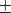 0,02	1,73 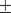 0,03	1,72 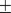 0,02
2,5%	1,55 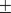 0,04	1,60 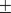 0,02	1,61 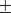 0,03	1,64 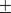 0,02	1,64 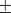 0,03
3%	1,49 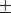 0,03	1,50 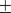 0,04	1,52 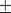 0,04	1,55 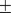 0,03	1,59 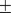 0,03

Dilution S1 contains
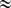
96 000 initial target copies per, S2
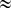
24 000, S3
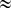
6000, S4
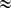
1500 and S5
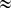
375.

Five point serial dilutions were created with a high number of repeats per dilution point (96 for the inhibitor free reactions, 48 for the inhibited reactions), starting at approximately 96 000 target copies and using four-fold dilution (initial target copies per reaction: S1
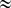
96 000, S2
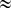
24 000, S3
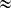
6000, S4
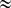
1500 and S5
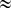
375).

All PCR reactions were performed in 25 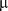
l using primers targeted against the soybean Lectin endogene (see table 2). The main reaction array was constructed using the Sltm primers only. SYBRgreen mastermix (Diagenode) was used with primers at a standard final concentration of 260 nM (1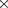), certain experiments used multiples of that standard concentration and are mentioned accordingly in the text (*e.g.* 4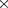 primer concentration means a concentration of 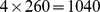 nM). All reactions were amplified in 96-well plates using a Biorad IQ5. A single protocol was used for all reactions: 10 min 95

C, 60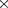 (15 sec 95

C, 1 min 60

C).

**Table 2:** Primer pairs used in this study.

Name	Sequence	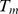	Length	Reference
Sltm1	5′-AACCGGTAGCGTTGCCAG-3′	59	81	[52]
Sltm2	5′-AGCCCATCTGCAAGCCTTT-3′	58,6		
Lec1	5′-CATCCACATTTGGGACAAAG-3′	54,1	96	[53]
Lec2	5′-TCTGCAAGCCTTTTTGTGTC-3′	56,2		
Lectin-F	5′-TCCACCCCCATCCACATTT-3′	55,8	81	[54]
Lectin-R	5′-GGCATAGAAGGTGAAGTTGAAGGA-3′	57,9		
GmaxLecFor	5′-CTTTCTCGCACCAATTGACA-3′	57,2	102	[55]
GmaxLecRev	5′-TCAAACTCAACAGCGACGAC-3′	60,2		
GM1-F	5′-CCAGCTTCGCCGCTTCCTTC-3′	63,3	74	[56]
GM1-R	5′-GAAGGCAAGCCCATCTGCAAGCC-3′	66,5		

Primer melting temperature is given under 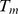 (as calculated by the wEMBOSS [57] program ‘dan’). ‘Length’ denotes the length of the amplicon in basepairs, ‘Reference’ indicates from which publication the respective primers were taken.

### Statistical Model

The data generating setup assumes that the evolution of a single reaction's efficiency over the different cycles behaves as a Gompertz type equation [32]. The double log of the cycle efficiency (ln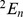) is modeled in function of the cycle fluorescence (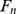) using an adaptation of the bilinear model from [33] as the efficiency decline has been observed to happen in two phases: an initial phase of gentle decline and a final phase of accelerated decline where fluorescence approaches its plateau.
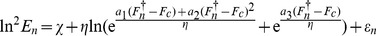
(1)


**Figure 1:**
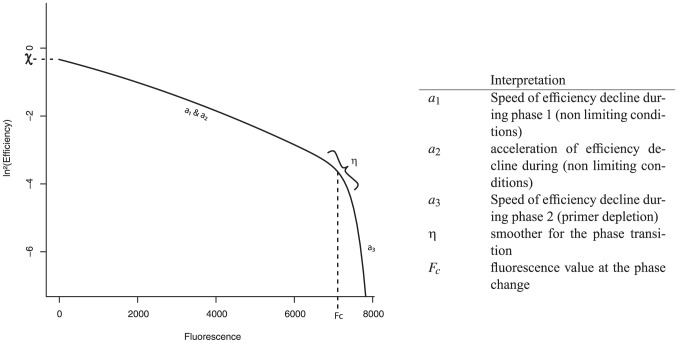
Illustration of the function of each of the six parameters of the bilinear model. 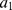
 and 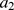 together describe the curve of the first phase, 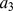 describes the slope of the second phase, 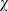 determines the 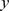-axis intercept (the intercept itself is ln^2^(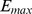)), 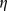 controls the abruptness of transition between the two phases and 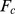 corresponds to the horizontal (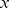-axis) position of the phase-change. The table on the right provides an overview of the physical interpretation of the model parameters.

The systematic part of the bilinear model (equation 1) takes six parameters: three ‘slopes’ (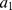 and 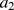 which together describe the curve of the first phase and 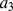 describing the slope of the second phase), a constant (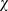) for shifting along the vertical axis, a parameter (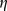) for adjusting the abruptness of transition between the two phases and a constant (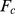) corresponding to the horizontal (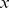-axis) position of the phase-change in efficiency decline (also see figure 1 for a graphical representation of the model parameters).

Parameter 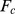 is the fluorescence value at which a first phase of gradual efficiency decline comes to a halt, when the reaction no longer sustains amplification due to primer depletion. Parameter 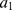 determines the slope of efficiency decline during this first phase and can be thought of as the speed with which efficiency initially proceeds to its minimum. Parameter 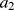 regulates the curvature of efficiency decline during this phase and can be thought of as the acceleration of the decline: the more curvature there is the more the decline speeds up over the course of the reaction. Parameter 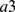 represents the steepness of decline during second phase of: the speed with which efficiency then drops to its minimum.

For the model to function as a data generating setup some modifications need to be made. Reaction efficiency is defined as the fold increase in target molecules after each cycle: 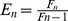 with both 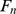 and 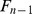 baseline subtracted fluorescence values [26], [34], [35]. Corollary, by definition, 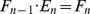. Thus, in order for the simulation to work sequentially, the bilinear model should be fitted by regressing ln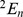 on 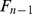, rather than on 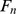 (as described in [26]), so that 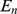 may be calculated from 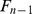. This yields:
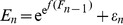
(2)where 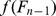 represents a function of 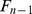. The obtained chain of cycle efficiencies can subsequently be converted to fluorescence values using the following equation of PCR kinetics:
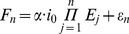
(3)where 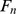 is the total amplicon fluorescence of cycle 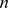, 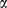 is the fluorescence emitted by a single amplicon, 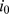 is the initial amount of target copies and 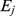 is the reaction efficiency of cycle 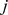. For the application of Gompertz curves in the direct modeling of reaction fluorescence see [36].

### Origins of variation

The goal of the data generating model is not only to simulate the systematic outcome of a given reaction setup, but also to investigate the variation between cycles of single reactions and between repeats of a single reaction. There are several possible sources of variation involved in the PCR amplification process even when, from the point of the experimenter, the initial conditions of template input and inhibition are fixed:

#### Initial copy variation

This is perhaps the most obvious source of variation between repeats. Differences in the number of initial target copies between repeats mainly arise from pipetting errors and the stochastic distribution of low concentrations of target molecules. Assuming that the target sequences are evenly distributed in the solution, the probability of a certain number of molecules pipetted into a reaction can be modeled by a Poisson distribution [37], [38].

#### Maximal efficiency variation

The between-repeat standard deviation (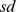) of the efficiency estimates in our dataset is about 0.025 (or 2.5% efficiency), but is larger in the case of inhibition (*e.g.* 0.068 or 6.8% when 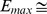
1.63). However, true variation in maximal efficiency between repeats is suspected to be much lower: the observed between-repeat variance is the sum of the variance on the estimates and the variance on the ‘true’ 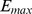. The former can be estimated using a bootstrap approach and is of the same order of magnitude as the estimated between repeat variance (standard deviation of about 0.03 when no inhibition is present). This indicates that the true variability of 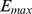 is likely to be very small (*i.e.* less then one percent). These findings are in line with results reported in [39] where the authors also conclude that variation in individually determined amplification efficiencies primarily represents random error and does not reflect true intra assay variation. In the simulation, random normal variation is used to generate differences in the true 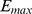.

#### Baseline

The level of base fluorescence may differ between repeats. The simulation uses a ‘modular’ approach to total fluorescence: it assumes base fluorescence change to be an independent parallel process, whose value is simply added to the amplicon fluorescence. This may very well be an oversimplification of the actual process, but the current level of the insight in the origin of base fluorescence does not support the development of an algorithm suitable for more accurate baseline simulation. A linear model is used, its values are seen as individual base fluorescence values for each cycle. Intercept and slope of the model are independently and randomly determined. Both are normally distributed with mean 0.7 and standard deviation 0.2 for the slope and with mean 200 and standard deviation 70 in case of the intercept (all values based upon empirical observations in the reaction database).

#### Camera noise

Almost all instruments display measurement error to some degree, a symmetric error term can thus be expected on the fluorescence measurement of every cycle within a reaction. Camera noise is simulated as additive error (normally distributed, standard deviation of 1.75 Fluorescence Units (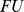) based on empirical observations).

### Data processing

All calculations and curve fitting were done using R version 2.13.0 [40]. The raw data were exported from the thermocycler and imported into R. Parameter modeling was accomplished using the standard linear modeling function (lm) in combination with nonlinear curve fitting using the Levenberg-Marquardt algorithm [41], [42] available through the package ‘minpack.lm’ version 1.1–5. The final simulation algorithm used in this publication is available as additional material and can be inspected for more detail on the exact methods used. See Algorithm S1.

### 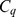 estimation


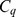
 values were estimated using two methods. **(I)** 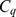 values are calculated as the cycle at which a fixed fluorescence threshold is reached for the baseline subtracted data. Interpolation is performed using the Forsythe, Malcolm and Moler spline [43]. **(II)** 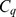 values are calculated as the position of the first positive maximum of the second derivative (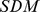) of a five parameter logistic model (5PLM) [44]:
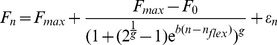
(4)where 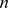 is the cycle number, 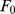 is the base fluorescence value, 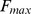 is the maximal fluorescence value which defines the plateau of the reaction, 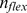 is the inflection point of the curve. Parameter 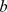 is the ‘growth rate’ and affects the slope of the curve at 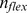 whereas 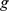 determines the asymptote where maximum growth occurs.

## Results and Discussion

In an initial step each separate reaction in the concentration-inhibition array of reactions (see materials and methods) was analyzed using the FPK-PCR approach. Efficiency estimates and bilinear model parameters were thus obtained, these estimates are treated mostly as close approximations of the true values: few aspects of their distribution are supposed to differ from the true parameter distribution.

We proceed by first discussing the distribution and properties of each model parameter. Second, the simulation of PCR reactions using parameter values drawn from these distributions is reviewed. Then, the addition of other sources of variation and their effect on the simulated curves is discussed. Finally, some aspects of the polymerase chain reaction are evaluated under the assumptions of the model.

### Parameter distributions

There are two aspects to consider: **(I)** the distribution of each parameter *per se* (for a given combination of 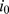 and 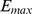) and **(II)** how the parameters change in response to a shift in either 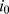 or 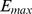 both jointly and separately (table 1 summarizes the combinations of 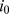 and 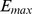). The former is limited to the observation that each distribution is symmetric and quasi normal. For the latter aspect, inspection of the physical meaning of each parameter helps to guide the interpretation of the observations.

The bilinear model has six parameters and each response in changes to both 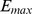 and 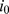 was examined. Some parameters were observed to be strongly affected by these changes (*i.e.* 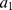, 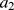 and 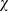). However, 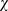 corresponds to the intercept of the bilinear model and can be obtained via a complex transformation of 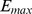, which itself is not explicitly present as a model parameter. Other parameters behaved more independently (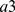, 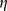 and 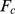), which is not surprising if we review their physical role (also see figure 1).

**Figure 2:**
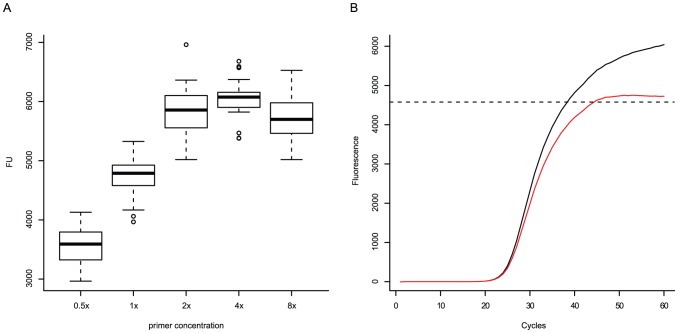
The effect of primer concentration. Panel **A** shows plateau values in response to changes in primer concentration (*Glycine max* Le1 gene at approx. 24000 copies, 24 repeats per primer concentration). Panel **B** shows PCR reactions (average baseline subtracted 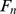 measurements over 24 repeats) of the same target (*Glycine max* Le1 gene) at approx. 24000 copies. The black reaction uses 8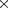 standard primer concentration (1040 nM) as opposed to the 1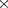 concentration of the red reaction (260 nM). The dashed line represents the calculated “ceiling” of the 1× primers reaction (*i.e.* 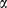 multiplied by the number of primers in the reaction).

Considering that parameter 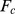 is the fluorescence value at which the transition from slower to rapid efficiency decline happens and taking into account that there are compelling indications that this second phase is caused by depletion of the primers in the reaction mix (see figure 2), it makes sense that the distribution of 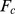 is constant with respect to changes in 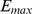 and 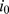. As all reactions have the same initial concentration of primers it takes the same number of amplicons to deplete each reaction's supply. Corollary, every reaction starts its second phase of decline at approximately the same baseline subtracted fluorescence value.

The distribution of parameter 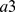 is also constant with respect to changes in 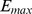 and 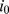. Indeed, as the second phase of decline is supposed to stand for efficiency decline under primer depletion, its value can be expected to be relatively constant. 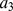 can be seen as the speed with which efficiency drops to its minimum when there are no more primers to sustain any form of amplification.

In summary, the near absence of response in changes to 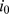 is consistent with the concept that the efficiency is predominantly a function of the concentration of reagents and reaction products and that other processes contribute only marginally to the main mode of efficiency attenuation. Essentially this means that for a given 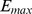 reactions should have identical ln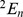 versus 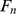 profiles whereas the number of cycles it takes to reach a certain fluorescence threshold would only be determined by its initial amplicon fluorescence (*i.e.* its initial target copy count as 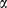 is presumed constant).

**Figure 3:**
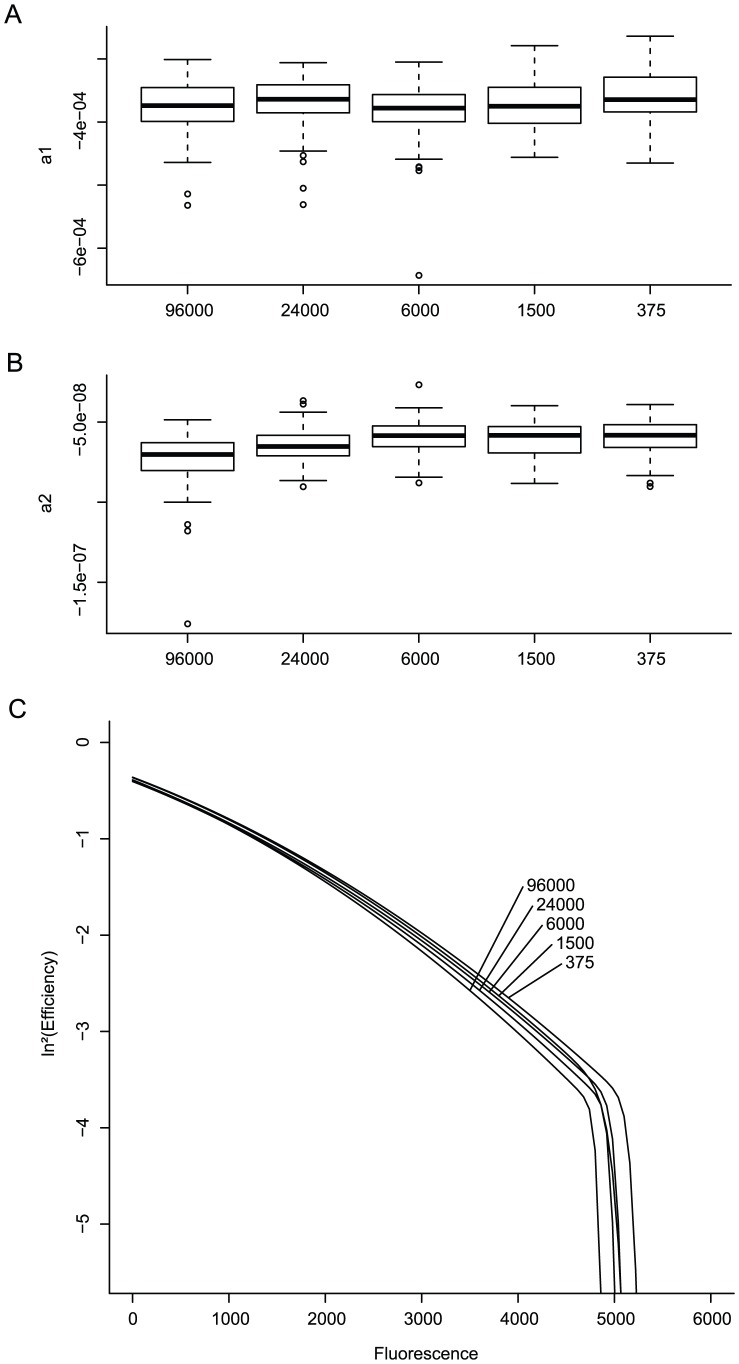
Variation of estimated parameters 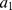
 **and** 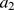 **in response to changes in** 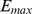 **(** ***i.e.*** **changes in inhibitor concentration).** Panels **A** and **B** show box and whiskers plots for the values of 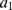 and 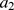 as estimated from the dataset. Each boxplot corresponds to repeats with the same concentration of inhibitor and maximal number of initial target copies (96 000). Panel **C** shows the resulting bilinear profiles, the reactions with highest inhibitor concentrations do not reach the plateau within the 60-cycle range (solid line) their theoretical continuation is shown as a dotted line in order to illustrate their general trend.

In response to changes in initial efficiency (*i.e.* increasing amounts of inhibitor) the values for 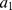 and 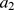 show a clear trend (figure 3, panels A and B). Parameter 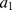 decreases as 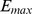 reaches lower values: the overall attenuation of efficiency proceeds faster when the initial efficiency is lower. Parameter 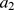 on the other hand increases from negative values for high values or 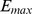 to positive values for low levels of initial efficiency: the curvature of the efficiency decline shifts from convex over straight to concave (figure 3, panel C). This means that, at least for isopropanol inhibition, the efficiency of reactions with a high 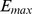 declines first slowly and then more rapidly, while for low values of 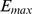 this behavior is reversed. Also note that due to the slower accumulation of amplicons the more inhibited reactions do not reach the point of primer depletion during the 60 cycles of the reaction.

Mathematically, parameter 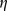 governs the speed of transition between the two phases. This transition is more difficult to fit so the amount of measurement error on this parameter is expected to be elevated. The fact that its value does not significantly change in response to differences in either 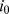 or 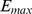 confirms that it takes a certain concentration or primer-to-template ratio for the polymerization to stall due to lack of primers, and that this ratio is fairly constant.

**Figure 4:**
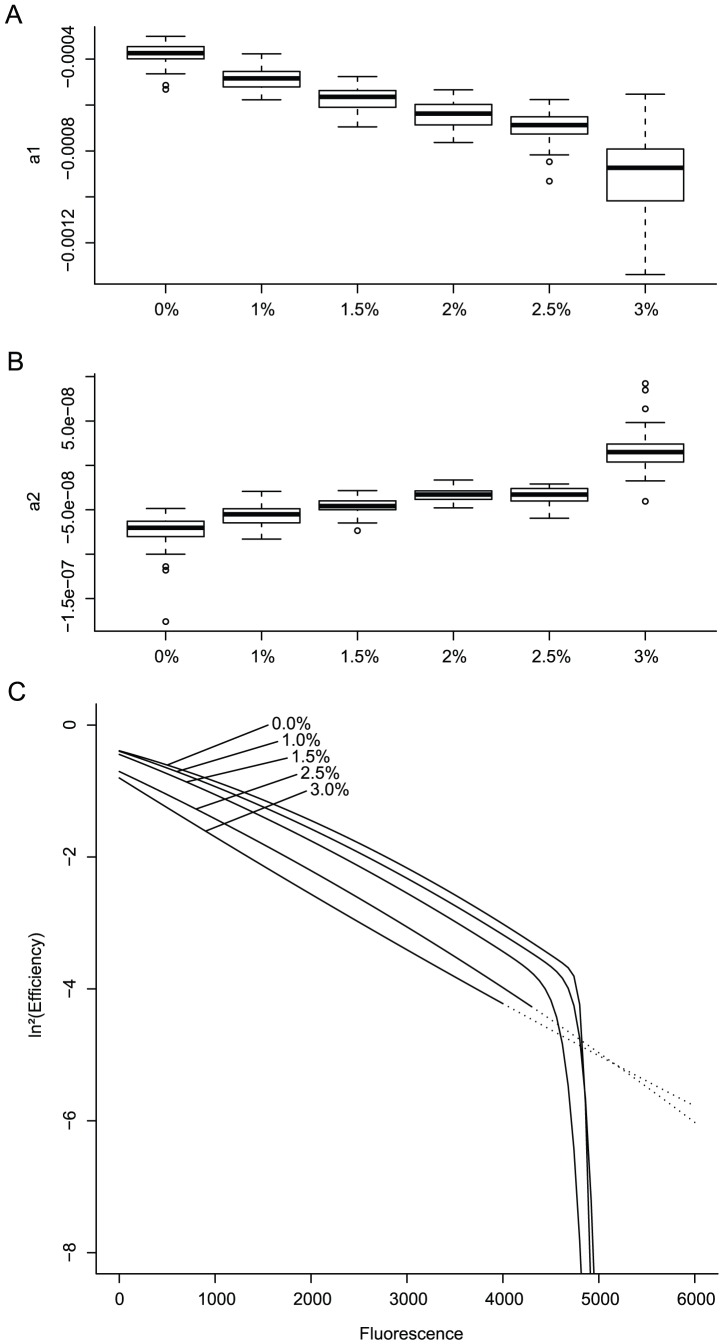
Variation of estimated parameters 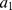
 **and** 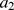 **in response to changes in** 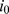 **.** Panels **A** and **B** show box and whiskers plots for the values of 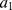 and 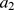 as estimated from the datasets. Each boxplot corresponds to repeats with the same number of initial target copies and maximal initial efficiency (no inhibitor present). Panel **C** shows the resulting bilinear profiles.

This leaves only two parameters (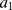 and 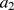) which determine most of the efficiency behavior. First we investigate their response to changes in 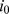. As can be seen from figure 4 panels A and B, the median values of both 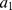 and 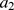 vary little over nearly three orders of magnitude in initial target copies. Indeed, no significant difference was found between the mean 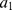 values of each dilution. For 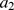 on the other hand, significant differences were found but a pairwise t-test showed that, in fact, only the two highest concentrations (*i.e.* 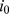 = 96 000 and 24 000) differ significantly from both each other and the rest. As a consequence we cannot rule out that this shift in mean 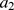 value is caused by unspecific amplification: reactions with low 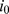 suffer from a more than proportional increase in fluorescence leading to an overestimation in 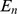 during the later cycles (figure 4, panel C). This makes sense as reactions with high initial copy numbers have a numerical advantage over any possible side processes when it comes to competition for reagents.

#### Joint parameter distribution

**Figure 5:**
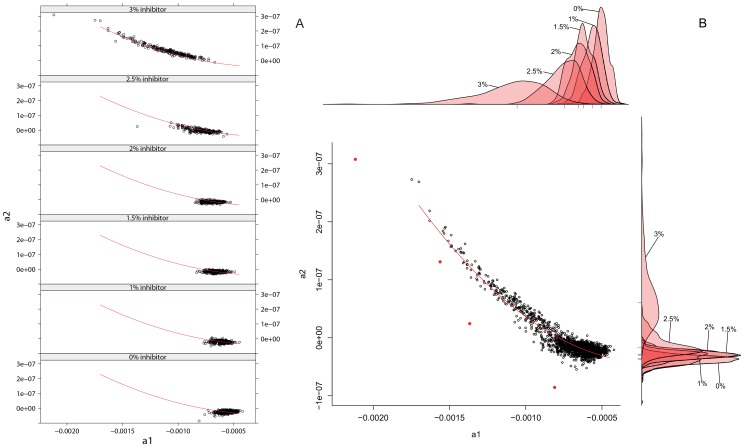
Scatterplot of estimates of parameters 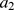
 **and** 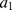 **.** Panel **A** shows separate scatterplots per inhibitor level whereas Panel **B** includes all available data (*i.e.* data for all copy numbers and all inhibitor concentrations) with the strongest outliers marked in red. Above and right of panel B the density plots per inhibitor level are shown. The tick marks beneath the density plots represent the median value per inhibitor level.

There is considerable covariance between the estimates of parameters 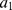 and 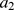 (spearman correlation: 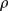 = −0,698) thus they cannot be considered independent for simulation purposes. As figure 5 illustrates the estimated values of 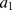 and 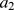 show a systematic non-linear association. Most of this effect is likely due to their mutual changes in response to increased levels of inhibition. Spearman correlations [45] between the variables 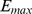-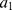-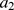 (see table 3) suggest a stronger linear association between 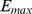-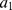 than between 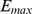-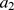 (also see figure 5), suggesting that the initial efficiency (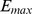) determines the overall speed of efficiency decline (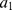), while the acceleration of the decline (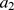, curvature) changes more in function of 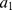 rather than 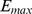. Indeed, incorporating 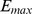 as a parameter in the 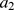 on 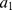 regression did not result in a better model (data not shown).

**Table 3:** Spearman correlation coefficients for the parameter estimates of 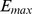, 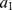 and 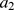.

Pairwise correlation			
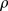	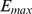	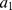	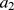
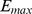	1,000	0,814	−0,640
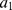	0,814	1,000	−0,698
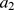	−0,640	−0,698	1,000

#### Limit of a reaction

As the number of initial target copies is known for each reaction in our dataset, it is possible to calculate the amount of amplicons that have accumulated at the phase change (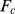): since the FPK-PCR analysis returns an estimate of 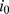 in terms of 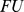 (*i.e.* 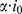, its product with the single amplicon fluorescence) one can calculate 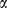 by dividing this estimate by the known template input (see table 4).

**Table 4:** Estimated single amplicon fluorescence for a number of PCR methods targeting the *Glycine max* Le1 gene (average estimate 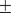 standard deviation).

primer	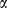	copy limit
Sltm	1,17 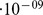 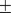 3,10 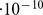	5,25 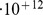
Lec	9,64 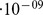 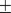 3,01 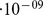	5,44 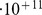
GMaxLec	6,96 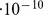 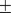 2,42 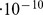	7,69 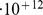
Pauli	9,08 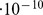 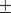 2,60 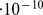	3,08 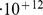
GM1	2,44 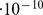 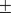 4,23 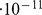	1,11 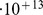

Their approximate maximum attainable copy numbers are also given (as estimated from their plateau value under 4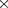 standard primer concentration).

With 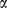 known, any baseline subtracted fluorescence value can be readily transformed into a number of template copies. This yielded an average of 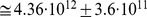 copies at 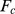 (mean 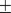 standard deviation), which is remarkably close to the total number of primers initially present in the reaction (260 nM in 25 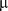
l yields 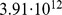 primers per reaction). Indeed, 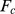 can be changed by changing the primer concentration (figure 2 panel A) suggesting that the onset of the second phase of efficiency decline is indeed caused by depletion of the primers.

In the original FPK-PCR publication the attenuation of efficiency was described to take place in two phases [26]. However, these findings now suggest that the second phase may not always be present (*i.e.* only in the case of reagent depletion). Indeed when running the reaction with an excess of primers (4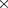 the standard concentration) the second phase does never occur and the reaction dies out more slowly under the influence of other processes (see figure 2 panel B). In such cases the complex bilinear equation model (equation 1) can be exchanged for a much simpler single phase equivalent:
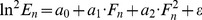
(5)


This indicates that, when a PCR reaction does not hit the hard limit of reagent depletion, it is essentially self limiting. The results from an experiment in which the primer concentration was varied between 1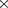 and 8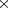 the standard concentration seem to agree with this concept. When primer conditions are not limiting, further increasing their concentration does not appear to shift the plateau accordingly (see figure 2 panel A).

When inspecting different primer pairs for the same target (see table 4 it is notable that the primer pair that produces the highest number of template copies (*i.e.* GM1) also has the highest primer melting temperatures (see table 2). It is indeed likely that the maximal attainable copy number (self limiting conditions) of a primer pair is determined by a combination of amplicon characteristics and primer attributes, *e.g.* melting temperature, amplicon length, GC content, etc.

### Simulation Engine

The main purpose of the simulation is to explore plausible origins of variation between repeats and their impact on the observed dispersion in fluorescence; it will also allow us to investigate certain aspects of the PCR reaction (*e.g.* length of the initial phase of maximal efficiency). The core of the data generating setup predicts the systematic outcome of a reaction based on the initial amount of target sequences and the initial efficiency. Subsequently, variation is introduced at several levels to obtain differences in cycle fluorescence and plateau level between repeats. Resulting amplification curves should be representative for observations in the data set.

**Figure 6:**
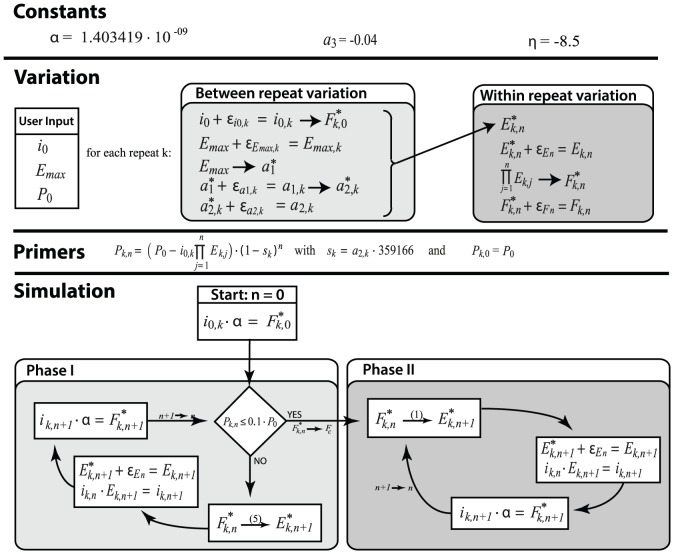
Schematic representation of the simulation process. The **upper panel** of the figure represents the error structure of the model as discussed under ‘evaluation of the sources of variation’. Arrows represent deterministic relations whereas 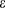 represents the introduction of random variation (represented as an additive process for the sake of simplicity). The **middle panel** of the figure illustrates how the number of primers in cycle n (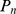) is calculated from the initial number of primers (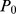) using the cycle efficiencies (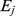) and the loss due to side processes (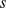). The **lower panel** of the figure represents the sequential application of the mathematical model. Within each phase the simulation repeats the same three steps: (**1**) the number of template copies accumulated during the 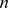 previous cycles (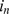) is converted to fluorescence (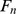) by multiplication with 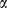. (**2**) the fluorescence level yields the efficiency by which the template will by duplicated during the current cycle (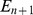) by using either equation 5 or 1 depending on the phase. In step (**3**) the actual amplification takes place: 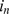 is multiplied by 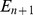 yielding 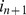. This marks the end of the (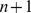)th cycle.

For the systematic part, simulation of real time PCR reactions can be reached by sequential application of the mathematical model. The simulation process starts with the initial number of target sequences (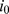) and the single amplicon fluorescence (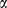). Their product (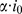) equals the initial amplicon fluorescence or 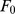. Using equation 5 the initial efficiency value (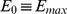) can be calculated from 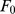. Since 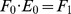 we advance one cycle. By iterating this process fluorescence values for every cycle can be obtained (see figure 6 for a schematic overview of the process).

**Figure 7:**
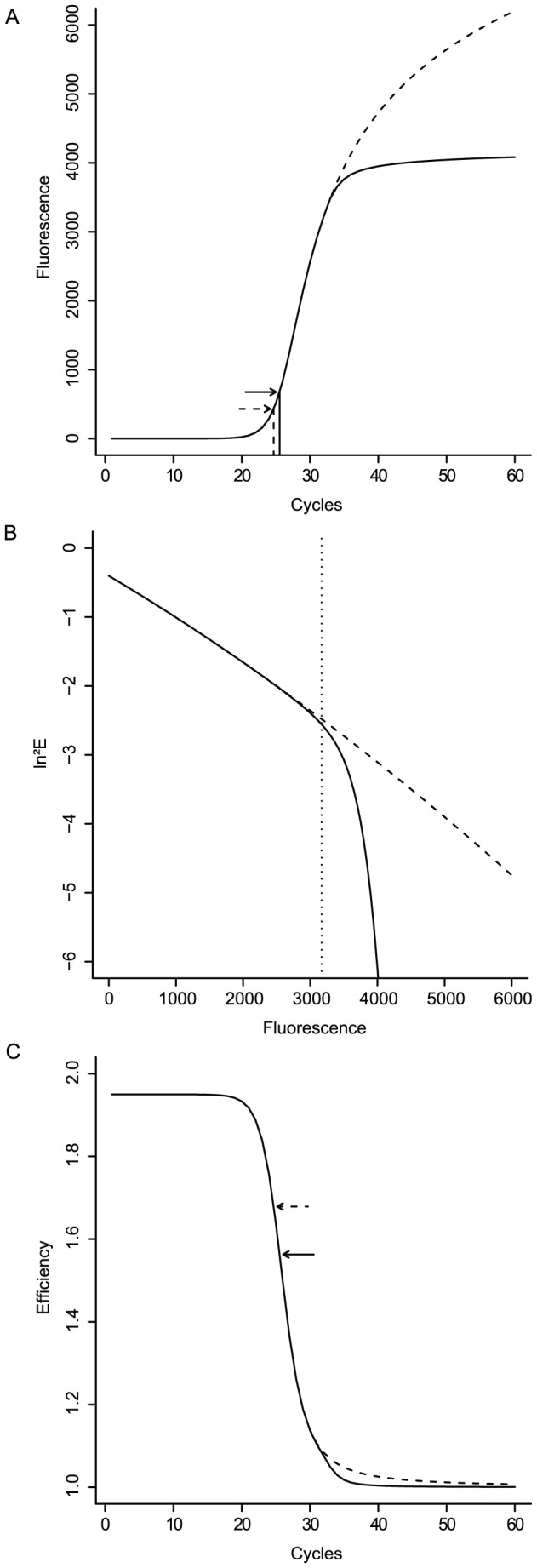
Simulation of the systematic outcome of a reaction. The simulation starts from 50 000 initial target copies and 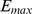 = 1.95. The results are shown in various representations: fluorescence versus cycle (panel **A**), ln^2^(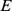) versus fluorescence (panel **B**), and efficiency versus cycle (panel **C**). The dashed line represents self limiting conditions, the solid line represents primer depleting conditions. The vertical dotted line in panel **A** represents the phase switch criterion: 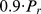. The two arrows in panel **C** mark the position of the second derivative maxima, the arrows in panel **B** mark the corresponding positions in the efficiency vs. cycle plot.

To take into account the possibility of primer limiting conditions the simulation has been divided into two independent modules or phases (see figure 6) which model the fluorescence path over two modes of amplification decline: self limiting (phase I) or primer depletion (phase II). The switch from phase I to phase II is governed by the concentration of primers (which is updated after every cycle). When 90% of the initial primers have been consumed transition to the second phase is initiated. This percentage was emperically found (data not shown) and produces simulation results in close approximation with the observations from the dataset (*i.e.* plateau level). Figure 7 demonstrates the results of switching from equation 5 to equation 1.

Before a simulation can start the model has to be populated with parameters. Only 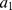 and 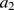 need to be determined in function of the simulation's starting conditions (*i.e.* 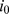 and 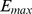), 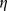 and 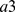 are constants (based on their estimated values in the real data; −8.5 and −0.04 respectively), 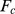 is the fluorescence value of the cycle in which 90% of all primers are consumed and is determined on the fly. The joint distribution of the parameters is most usefully decomposed in the following order: 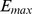 (user input), next 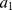 is found using equation 6 and finally 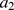 follows from equation 7. Both equations were determined by regressing the parameter values observed in the reaction dataset taking into account the heteroskedastic nature of the error structure (weighted least squares). Note that 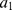 and 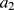 are assumed to be independent of 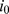: the initial efficiency determines the type of decline curve whereas the initial number of target copies determines at which position of the curve the reaction starts (also see figure S1 in the supplemental material).
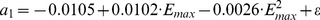
(6)

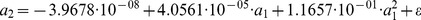
(7)


When primers are not limiting the simulated amplification curves show a gradual transition from linear amplification to plateau phase, resulting in a “round” or obtuse amplification profile (*e.g.* the dashed line in panel A of figure 7). In case of primer depletion the reaction is suddenly stopped over the course of a few cycles as primer concentration reaches critical values. As a result, the simulated fluorescence values have a more “angular” or acute profile, depending on the stage of the reaction when the primers become limiting (*e.g.* the solid line in panel A of figure 7).

Panel B and C of the same figure further illustrate both scenarios: in bilinear form (ln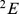 vs. fluorescence, panel B), and in more standard form (efficiency vs. cycle, panel C). The differences between primer depletion and self limiting conditions are most obvious from panels A and B, while the standard efficiency vs. cycle plot (C) illustrates how relatively small differences in cycle efficiency have a strong impact on the reaction's overall profile due to the cumulative nature of the amplification process.

### Evaluation of the sources of variation

For the simulation model to be deemed plausible, its observable consequences should match what is seen in the data. Four elements were considered when evaluating the variation patterns of the simulated reactions: **(I)** for any given initial number of target copies the 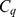 values should be close to the respective values observed in the dataset, **(II)** the 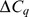 between two simulations with a different number of initial targets should be very close to its theoretical value taking into account the input 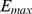, **(III)** the spread of 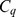 values between repeated simulations should approximate the spread observed in the dataset and **(IV)** the spread of the fluorescence plateau between repeated simulations should also approximate the spread observed in the dataset.

Of these four elements, the first two (acceptable 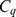 and 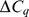) are embedded in the model for the systematic outcome of the reaction and did not pose any problem: none of the tested combinations of 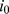 and 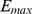 resulted in simulated 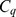 values that were either far from the observations in the dataset or incorrectly spaced with regard to the initial number of target copies. The two other criteria are discussed per source of variation:

#### Baseline variation

Since variation of the baseline is considered in a purely additive form, there are only minimal differences in plateau level when adding baseline variation alone to the simulated reactions and there is no kinetic variation. The resulting dispersion in 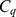 values is very small indeed (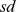 = 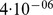), as is the dispersion of the plateau values (coefficient of variation: 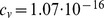 after baseline subtraction). Hence, baseline variation does not explain the actual variation seen in plateau levels.

#### Camera noise

On its own, as sole source of variation, camera noise adds little plateau differentiation (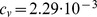), the standard deviation of the 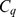 values is 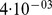.

#### 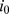 variation

At high numbers of initial target copies (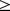
50 000) the variation introduced through the Poisson distribution into the amplification curves is minimal in both plateau level (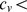
0.005) and 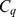 estimates (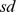 = 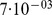). When lowering the copy number, the contributed variation becomes more considerable (at 500 copies 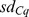 = 0.05; at 50 copies the plateau 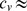
0.01 and 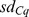 = 0.2). However, the standard deviation of the 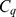 values in the dataset is on average 0.12 (without inhibition) and the plateau 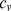 is about 0.09. This indicates that only a small percentage of the total variation witnessed in 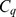 and plateau level may be due to 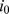 differences between repeats.

#### 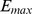 variation

Of all four sources of variation tested, this is the only factor that introduces significant overall variation between the curves. Now however, the amount of diversity also rapidly increases in function of the variation added: with a true 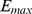 of 1.9 and 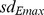 of 0.01 (1 percent of efficiency) the standard deviation of the 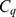 estimates is about 0.2 and 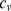 of the plateau is 0.012, at a true 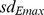 of 0.05 the 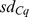 and 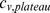 are about 0.9 and 0.012 respectively. At a true 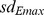 of 0.1 the 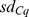 has increased to 1.75 whereas the 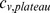 remains relatively constant (*i.e.* 0.011). In the observation dataset, the 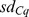 never exceeds 0.175 (for reactions without inhibition). Since the latter is the result of all sources of variation combined it is most likely that the true 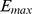 variation between repeats is below 1 percent of efficiency (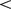
0.01). Thus, differences in initial efficiency between repeats does not seem likely as main cause of plateau variation.

None of these sources alone introduces diversity between repeats comparable to the observations in the dataset and neither does their cumulative effect. When all of the above are combined in an additive fashion, even though they do cause an amount of 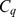 variation comparable to the dataset, there still is considerably less plateau variation in the simulated amplification curves (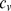 is 0.02 compared to the 0.09 in the dataset). Therefore, two further sources of variation were inspected: **(I)** random error on the cycle efficiency within a single reaction (departures from the theoretical 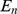 values), and **(II)** small differences in the profile of efficiency attenuation between repeats (departures from the theoretical 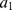 and 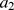 values). These two sources represent further kinetic differences between repeats besides differences in initial efficiency.

#### 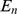 variation

Addition of random error with a constant standard deviation to every 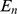 resulted in very unstable amplification curves. Instead, random error with a constant *relative* standard deviation was used. This way, the absolute deviation of the cycle efficiency from its theoretical value becomes smaller as efficiency declines. Even so, the addition of 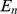 error could not produce the necessary plateau variation without resulting in overly unstable amplification profiles and inflated 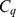 standard deviation. Therefore, such random error on 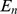 error is neither considered to be the main explanation of differences between the plateau levels of repeats.

#### 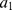
–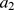 variation

This was found to be the only source of random variation that induces considerable differences between the curves and plateau levels of simulated repeats. However, it proved to be impossible to inflate the plateau variance without causing a large discrepancy in variation between 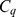 values as calculated using the 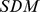 and using a standard threshold. Normally these two values are in close approximation of each other and their standard deviation is very similar. The 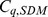 has been reported to be more stable than 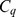 values calculated using a threshold [28], [46]. The 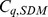 is based on parameters from the 5PLM (4) and its standard deviation is an indicator of the overall shape diversity between curves which is considered very stable. Indeed, parameter comparison has been successfully used for the detection of outlier reactions [47], [48]. Therefore, the simulated repeats' 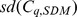 should not surpass the 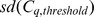 and the use of kinetic differences between repeats to drive plateau variation is not considered to contribute to a more realistic simulation of between reaction variation.

**Figure 8:**
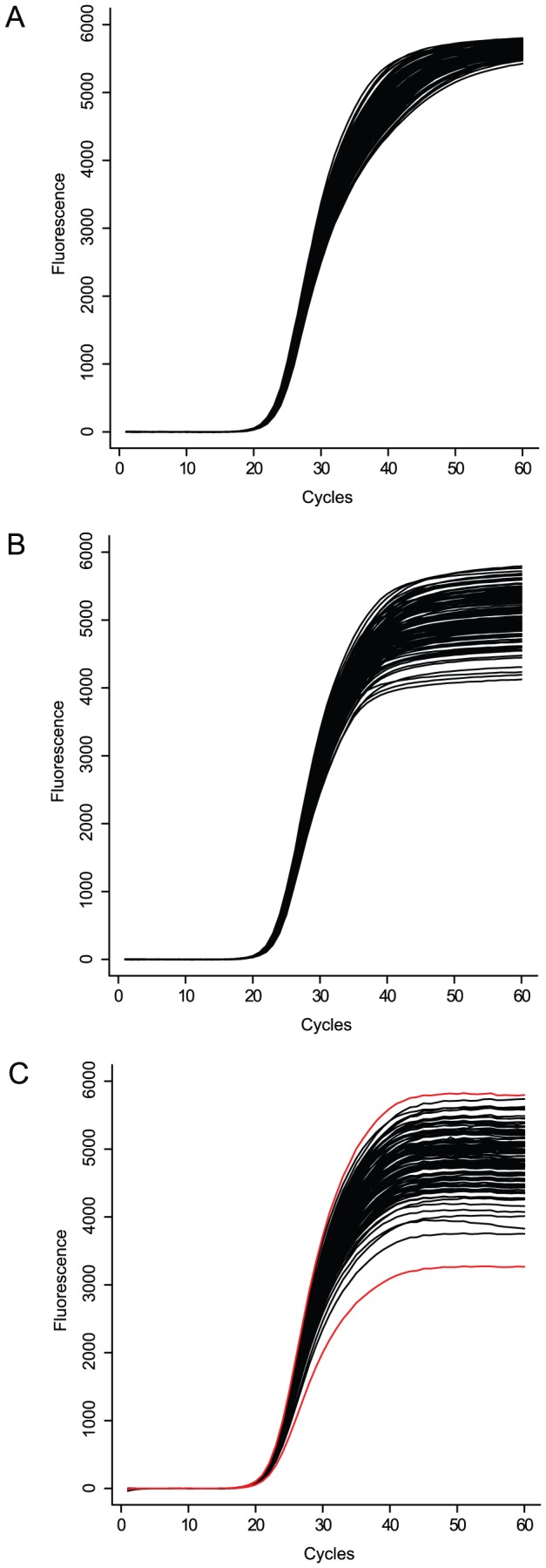
Observed and simulated between-repeat variation. Panels **A** and **B** show simulated repeats of a reaction with 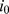 = 96 000, 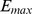 = 1.95. Panel **C** shows the baseline subtracted amplification curves of 96 actual repeats of a similar reaction (targeting the *Glycine max* Le1 gene at approximately 96000 copies, mean estimated 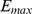 = 1.97). In panel **B** additional loss of primers due to unspecific processes has been simulated, whereas in panel **A** target amplification is the sole source of primer consumption.

In summary, the final result of all these variation sources combined does still not reproduce the observed dispersion in plateau levels (also see figure 8 panel A). The main reason behind the large variation in plateau levels thus appears to stem from differences between repeats in 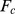, the point at which primers become limiting, rather than kinetic asymmetries. This either implies a large variation in primer concentration between the repeats, which is unlikely in view of the experimental setup, or a primer consumption that is not only driven by template amplification but also by side processes which differ among repeats.

The original simulation updates the current primer concentration after every cycle by subtracting the number of amplicons formed from the number of primers at the start of the cycle. Primer consuming side processes can now be simulated by further diminishing the primer concentration through subtraction with a fixed percentage of the current primer count. *i.e.* each cycle 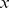
% of the primers available at the start of the cycle are lost to the side process (with 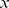 normally distributed around 2.27 with a standard deviation of 0.47). This indeed increased plateau variance significantly (see figure 8 panel B). However, a striking feature of the actual data is that the amplification curve that emerges first (lowest fractional 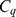) has the highest plateau level and *vice versa* (see the red lines in panel C, same figure). But when assigning side process greediness at random this relation is abandoned and the plateau-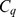 relation is randomized too. Indeed, there is an amount of correlation between the estimates of paramters 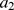 and 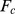 (correlation: 0.46) which has to be respected: the lowest 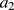 values should also have the lowest 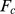 (*i.e.* the highest side reaction activity, see figure 6, middle panel) to obtain a similar result in the simulated repeats (see figure 8 panel B).

The model that is thus suggested by these observations is one where the efficiency decline is a strict function of the concentration in reagent and reaction products (one set of bilinear parameters for a given 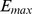, irrespective of 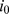.) whose profile is modulated by one or more side processes that bring about repeat-specific changes to the reaction kinetics through the additional consumption of reagents (variation in 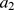 and 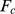) that add to the random variation inherently present in the PCR process (random error on 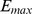, 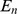 and 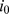, baseline variation, *etc.*).

Analysis of the outliers (figure 5B, red dots) supports this view. Reactions with outlying 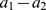 pairs indeed have outlying plateau levels (z-score on average
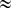
−4). Such low plateau levels could not be recreated using extreme 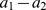 combinations alone. Only when combined with the corresponding levels of exceptional primer loss such outlying amplification profiles could be generated.

### Aspects of PCR

An achievement of the current model is that it reliably predicts the systematic outcome and variation within & between reactions given a set of 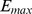 and 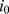 conditions. It can therefore be used to investigate a number of aspects of the PCR reaction and derived estimates that are inaccessible in real data. There is, however, no guarantee that the model components represent physical reality apart from their ability to simulate realistic patterns of observations as witnessed in the dataset.

#### Number of cycles with constant efficiency

The statistical model does not allow for a phase of truly constant efficiency, it rather contains a phase of ‘minimal decline’ during which the efficiency changes very little, followed by a period of rapid attenuation (see figure 7 panel B). To be able to calculate the length of the ‘exponential phase’ we will therefore consider the efficiency constant until the model reaches a decrease of 0.01 or one percent of efficiency with respect to its initial value. At 50 000 initial target copies and an 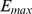 of 1.90 this point is reached during the 21st cycle (fractional cycle: 20.3). During the following two cycles, the efficiency begins to drop more rapidly (1.88 and 1.86 in cycles 21.3 and 22.3 respectively). For a reaction with those initial conditions, the FPK-PCR considers the ground phase to end by cycle 18 (*i.e.* the point at which amplicon fluorescence becomes discernible from the base fluorescence) and the approach published in [28] indicates fractional cycle 17.8 as the starting point of the exponential phase. These results indicate that the phase of constant efficiency may be drawing to its end by the time amplicon fluorescence can be distinguished from the background. This questions the existence of a true phase of exponential amplification in the data.

#### Second Derivative Maximum

Figure 7 panels B and C indicate the position of the 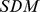 on the reaction curves, which is several cycles beyond the final cycle of constant efficiency (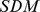 = 25.6 or 26.7 when primers are limiting). Due to its dependence on the form parameters of the 5PLM (4) its position is influenced by the primer conditions and does not *per se* correspond to a fixed moment in reaction kinetics. The exponential phase has indeed ended by the 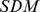 but using it as a marker to define a window of application for an exponential fit may lead to the inclusion of several cycles of decreased efficiency and ultimately to a biased efficiency estimate.

When inspecting the position of the 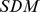 for different values of 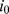 we noted that the lower the initial copy number, the higher the 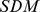 is situated on the amplification curve. Due to the steepness of the amplification curve there is relatively little 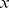-axis shift so that this displacement is not obvious from the 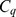 values, but it might suffice to bias the conclusions of an assay.

**Figure 9:**
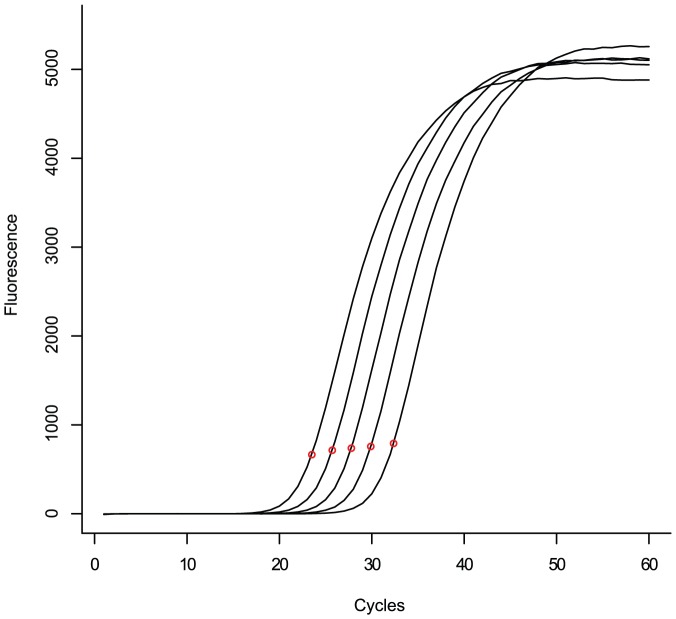
Upward displacement of the 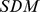
 **.** This figure illustrates the increase in 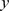-axis position of the 5PLM second derivative maximum with decreasing 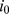. The amplification curves are the average fluorescence measurements of a *Glycine max* dilution series (96 repeats per dilution point, Le1 gene target).

There is no strict mathematical ground for this effect: the 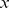-axis position of the inflection point (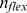) plays no role in the calculation of the 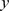-axis position of the 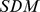. Therefore, the assumption that the growth parameters 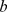 and 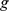 remain constant, not only between repeats of a single reaction but also over all values of 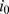, may not be entirely correct. Although first observed in the simulations, this upward displacement of the 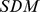 was confirmed in the reaction dataset (figure 9) as well as in other dilution series that used different template DNA and primers (data not shown). In this light, calculating 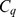 values using a fixed fluorescence threshold, for instance placed at the 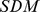 with the lowest 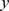-axis position, seems more appropriate than using each curve's individual 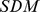 value.

#### FPK-PCR estimates

The statistical model behind the simulation engine is also the principle by which the FPK-PCR approach analyzes reactions and thus a certain amount of bias can be expected when using this for its evaluation. Nevertheless, inspection of its general performance on the detection of systematic effects is useful. For this purpose a twofold dilution series ranging from 100 000 down to 390 copies was simulated at an initial efficiency of 1.95 with all sources of variation present. The resulting set of 800 reactions was subsequently analyzed using the FPK-PCR algorithm presented in [26].

The FPK-PCR 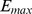 estimates are stable over the entire dilution series and were not affected by changes in input 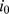. The dilution factor obtained from the 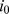 estimates is correct (*i.e.* 2.01). The 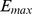 estimates were on average 1.997
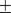
0.025 over all 800 reactions. This overestimation of efficiency is persistent with regard to changes in both input 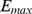 and input 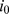. Corollary, these elevated efficiency estimates do not preclude their use in comparing reactions and the ability of the FPK-PCR approach to detect kinetic outliers is not compromised. However, the variation in the FPK-PCR initial copy number estimates is more than twice the variation in copy number estimates based on 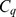 values (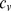 0.25 and 0.12 respectively). The FPK-PCR 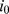 estimator relies heavily on the assumption that all changes in reaction fluorescence are due to the amplification process. Any alternative process that adds variation to the final observed fluorescence (i.e. plateau variability) thus translates into additional variation of these 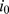 estimates. An advanced 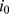 estimation method capable of discounting this extra source of variation is under development and one element of a planned update of the FPK-PCR algorithm.

These results for the FPK-PCR approach are in line with the findings from a recent comparison of real time PCR analysis methods (Ruijter *et al.*, in publication): slight overestimation of the initial efficiency and increased variability of the estimates of the number of initial target sequences. The study further acknowledges the FPK-PCR's suitability in detecting kinetic outliers (inhibition) and its performance on a complex biological dataset.

## Conclusions

To enable study of the variance of key estimates in a highly complex setting, we have developed a novel approach that does not merely simulate data from a postulated model. Our approach is designed to minimize the risk of missing true residual variation in the data, and we would like to coin the counterfactual ‘data’ involved: ‘Simurealizations’. These start from a well balanced, especially constructed dataset of observations, providing real responses in function of varying key input parameters. The data generating model is then adapted through various cycles of comparison to the real data. This allows stepwise addition of variance components to the model, until the resulting simulated data are close enough to reality from the perspective of the key targeted features in the analysis. The model can subsequently be used to evaluate results and properties from the original model fitting technique in this more complex setup. Such strategy could prove useful more generally in high dimensional arrangements.

In the present setup, starting from a relatively simple statistical model for the evolution of efficiency in a single PCR reaction we have added one error component at a time to arrive at a data generation setup for repeats which produces simulated data whose between- and within-reaction variation has realistic features. The outcome of the simulations is a realistic reproduction of the observations from a large dataset: The 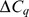 between reactions is accurate given the input 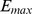, the size of the 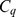 values with respect to the initial number of targets is in line with our observations, as is the spread of the 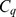 values.

The early stages of PCR reactions were found to be largely independent of primer and amplicon sequence. It seems, however, that this does not hold for the later stages of the reaction and the specifics of efficiency attenuation, in particular the self limiting properties of the reaction were found to differ between primer-pairs.

Most of the variation in 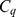 values could be adequately captured by the statistical model in terms of random error. However, to recreate a dispersion of plateau level equal to that in the reference dataset, while keeping the other aspects of the PCR curves within realistic bounds, additional sources of reagent consumption needed to enter the model. These results are consistent with an efficiency that behaves foremost as a function of the concentrations in reagents and reaction products, while the large variation in fluorescence between repeats during the later cycles is caused by differences in the amount of reagents lost to unspecific processes.

In order to arrive at simulations with a realistic dispersion of fluorescence among repeats, the true variation in initial efficiency had to be kept minimal. These findings are in accordance with among others [39], [49] where the authors indicate that sample specific efficiency correction increases the random error. Therefore, approaches like Kinetic Outlier Detection (KOD) [47], [48], [50], [51] seem the best strategy in using the efficiency estimates to ensure similarity of kinetics between reactions.

Little evidence could be found that the 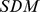 is an appropriate marker for the end of the exponential phase. Its increase in 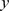-axis position with decreasing initial target copies may introduce bias when 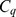 values are calculated at individual 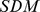 positions. It has also been shown that primer concentration may influence the position of the second derivative maximum on the amplification curve. While primer concentration is not likely to vary over repeats, it is a factor to keep in mind when using second derivative maxima in the kinetic analysis of PCR.

Based on these findings we are able to formulate a number of guidelines for minimizing between repeat variation in a qPCR setup. Firstly, the use of the 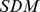 is discouraged **(A)** as a kinetic marker, as it may not always correspond to the same stage of reaction kinetics, and **(B)** to calculate 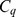 values for individual reactions. A classical ‘fixed’ threshold may be preferable in view of the latter. However, the 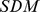 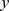-axis position is a useful criterion for selecting a user-independent threshold position (*e.g.* using the 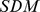 with the lowest 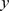-axis position in the reaction set). Second, we would like to stress the importance of minimizing side reactions when possible (*e.g.* through primer selection) in order to avoid excess variation between repeats. Finally, increasing the primer concentration and running additional cycles may help obtain more data for analyzing reaction kinetics with models like FPK-PCR and LRE.

Further use can be derived from the simulation engine: by adjusting key parameters it can be tailored to emulate specific reactions. This allows then to gauge the amount of variation that can be expected under certain conditions of 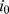 and 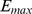. The presented results serve as input for future design of PCR analysis methods or the improvement of existing approaches. A better captation of sources of variation in the data leads to an improved distinction between signal and noise and hence diminishes bias and increases precision. This may ultimately allow to control the risk of claiming absence of particular DNA species in settings where such detection is of prime importance.

In summary, we developed a simulation tool that proved to be useful in evaluating reliability and precision of qPCR results. It allowed us to discover hitherto unrecognized sources of error and propose method improvements accordingly. As it stands, the approach can be quite generally used and, if needed, naturally adapted to new settings.

## Supporting Information

Figure S1
**Scatterplot of estimates of parameters** 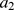 **and** 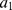 **.** Separate subplots per level of initial target copies are shown, each subplot contains data from all levels of inhibitor concentration. In each subplot the curve of equation 7 is shown as a red line. These plots indicate that the assumption that 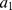 and 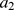 are independent of 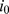 (and solely dependent on the initial efficiency) is justified.(EPS)

Algorithm S1
**The algorithm provided (generator_v6X_15.r) is written in R, a free software environment for statistical computing and graphics (** http://www.r-project.org/ **).** The file is intended to be loaded as ‘source R code’ into the algorithm and contains a single function (generate.pcr()) with the following arguments:mu.i: numerical. The desired average initial targets per reaction.output: character. The type of algorithm output to be returned: “i” for cycle target copies, “e” for cycle efficiencies, “f” for cycle fluorescence values or “p” for the bilinear model parameters (default is “i”).Emax: numerical. The desired initial reaction efficiency (default is 1.95).cycles: numerical. The desired number of PCR cycles (default is 60).primers: numerical. The desired primer concentration in the final reaction volume, in 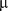
M (default is 260).vol: numerical. The desired reaction volume in 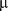
L (default is 25).plots: logical. If true, the plots are produced that visualize the output (default is FALSE).variation: The desired types of variation to be used in the simulation process. Its value shoudl be either 0 or “E” for 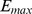 variation, “En” for 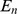 random error, “i” for 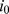 variation (pipetting error), “p” for primer variation, “s” for side reactions, “a” for kinetic variation or a vector with any combination of these (e.g. c(“E”,“i”)). Default is c (“En”, “E”, “i”, “p”, “s”, “a”).baseline: logical. If true, a random baseline is added to each generated curve (default is FALSE)Cq: logical. If true, an additional 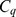 analysis is performed on the simulated reactions and the results are reported (Default is FALSE).
**Output**A matrix of 100 columns and as many rows as there are cycles in the simulation (default is 60). Each column contains a single simulated reaction. The actual output depends on the user input (argument output): if “i” was specified the number of amplicons present at the end of each cycle is returned, if “e” was specified the efficiency value of each cycle is returned, if “f” was specified the fluorescence values are returned, and if “p” was specified the bilinear model parameters for each simulated reaction are returned yielding a 6 by 100 matrix. By default the amplicon accumulation is returned.**Examples**## A minimal function call generate.pcr(10000)## Producing graphical output generate.pcr(15000, plots = T)## Fluorescence output with baseline added and using only pipetting error generate.pcr (20000,variation = “i”,baseline = T, plots = T).(R)
